# *Prunus armeniaca* gum exudates: An overview on purification, structure, physicochemical properties, and applications

**DOI:** 10.1002/fsn3.2107

**Published:** 2021-01-08

**Authors:** Davoud Salarbashi, Kambiz Jahanbin, Mohsen Tafaghodi, Elham Fahmideh‐Rad

**Affiliations:** ^1^ Nanomedicine Research Center Gonabad University of Medical Sciences Gonabad Iran; ^2^ Department of Food science and Nutrition School of Medicine Gonabad University of Medical Sciences Gonabad Iran; ^3^ Department of Food Science and Technology Faculty of Agriculture Shahrood University of Technology Shahrood Iran; ^4^ Nanotechnology Research Center Pharmaceutical Technology Institute Mashhad University of Medical Sciences Mashhad Iran; ^5^ Pharmaceutics Department School of Pharmacy Mashhad University of Medical Sciences Mashhad Iran; ^6^ Applied Sciences Department, Applied Chemistry Section Higher College of Technology (HCT) Muscat Sultanate of Oman

**Keywords:** food and pharmaceutical application, physicochemical properties, *Prunus armeniaca* gum exudate

## Abstract

*Prunus armeniaca* gum exudate (PAGE) is obtained from the trunk branches of apricot trees. PAGE is a high‐molecular‐weight polysaccharide with arabinogalactan structure. The physicochemical and rheological characteristics of this gum have been investigated in various researches. PAGE offers a good potential for use as an emulsifying, binding, and stabilizing agent in food and pharmaceutical industries. It also can be used as an organic additive in tissue culture media, synthesizing of metallic nanoparticles, binding potential in tablets, antioxidant agent, and corrosion inhibitor. For desirable emulsifying, stabilizing, shelf life‐enhancing properties, and antioxidant activity of PAGE, it can be used as additive in many foods. We present here a comprehensive review on the existing literatures on characterization of this source of polysaccharide to explore its potential applications in various systems.

## Graphical abstract

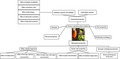

## 1 INTRODUCTION

Hydrocolloids are broadly employed in the food industry for several aims, such as edible coatings, thickener and stabilizing agents, carrier, and as an emulsifying agent (Maeda et al., 2000). In traditional medicine, gum exudates are widely utilized for the treatment of various diseases caused by oxidative damage (Wu et al., 2012) such as anticancer (Chen et al., 2013; Jeff et al., 2013), immunomodulation (Zhang et al., 2010), antioxidant, and anti‐inflammatory properties (Ananthi et al., 2010; Wu et al., 2012). Furthermore, these hydrocolloids could show extensive biological activities including antioxidant, antitumor, and anti‐inflammatory activities (Cho et al., 2000; Hui et al., 2006; Malsawmtluangi et al., 2014; Xia et al., 2011; Xie et al., 2013). Due to the wide range of hydrocolloids applications in different industries, the search for novel source of natural polymers has attracted the attention of researchers in recent years (Warrand, 2006).

*Prunus armeniaca* L. belongs to Rosaceae family and is widely distributed in Asia (Hormaza, 2002). The gum exudate obtained from the branches of *P. armeniaca* trees is used as a food additive (Nussinovitch, 2009). For instance, in Iran, this gum is used for the treatment of cough, and the improvement of complexion and eyesight (Zargari, 1994).

Since the functional and physicochemical characteristics of the hydrocolloids are mainly dependent on their chemical composition as well as structural properties (Sherahi et al., 2017), it is necessary to determine the chemical composition and structural properties of the new source of hydrocolloids. Various studies have been conducted to characterize *Prunus* family gum exudates (PAGE) and evaluate its physicochemical, structural, and functional properties (Al‐idee et al., 2020; Alwaan & Mahdi, 2016; Azam Khan et al., 2012; Babken et al., 2018; Bouaziz et al., 2015; Chichoyan, 2015; Chichoyan & Shaboyan, 2016; Ding et al., 2019; Ergin et al., 2018; Fathi, Mohebbi, & Koocheki, 2016a, 2016b; Fathi et al., 2017; Hamdani et al., 2017, 2018; Hashemi & Raeisi, 2018; Islam et al., 2019; Jamila et al., 2020; Khorsha et al., 2016; Kora, 2018; Licá et al., 2018; Lluveras‐Tenorio et al., 2012; Molaei & Jahanbin, 2018; Najafi et al., 2020; Rahim et al., 2018; Shamsara et al., 2015, 2017; Shi et al., 2019; Stephen & Shirley, 1986; Theophil Anand et al., 2019; Wei, Zhang, He, et al., 2019; Wei, Zhang, Zhang, et al., 2019; Zhang et al., 2020). We tried to present a compressive review on the documents on PAGE to explore its potential application in food, textile, cosmetic, and pharmaceutical systems. Furthermore, the relationship between physicochemical and functional properties of PAGE was discussed in the present review.

## 2 PURIFICATION

**FIGURE 1:**
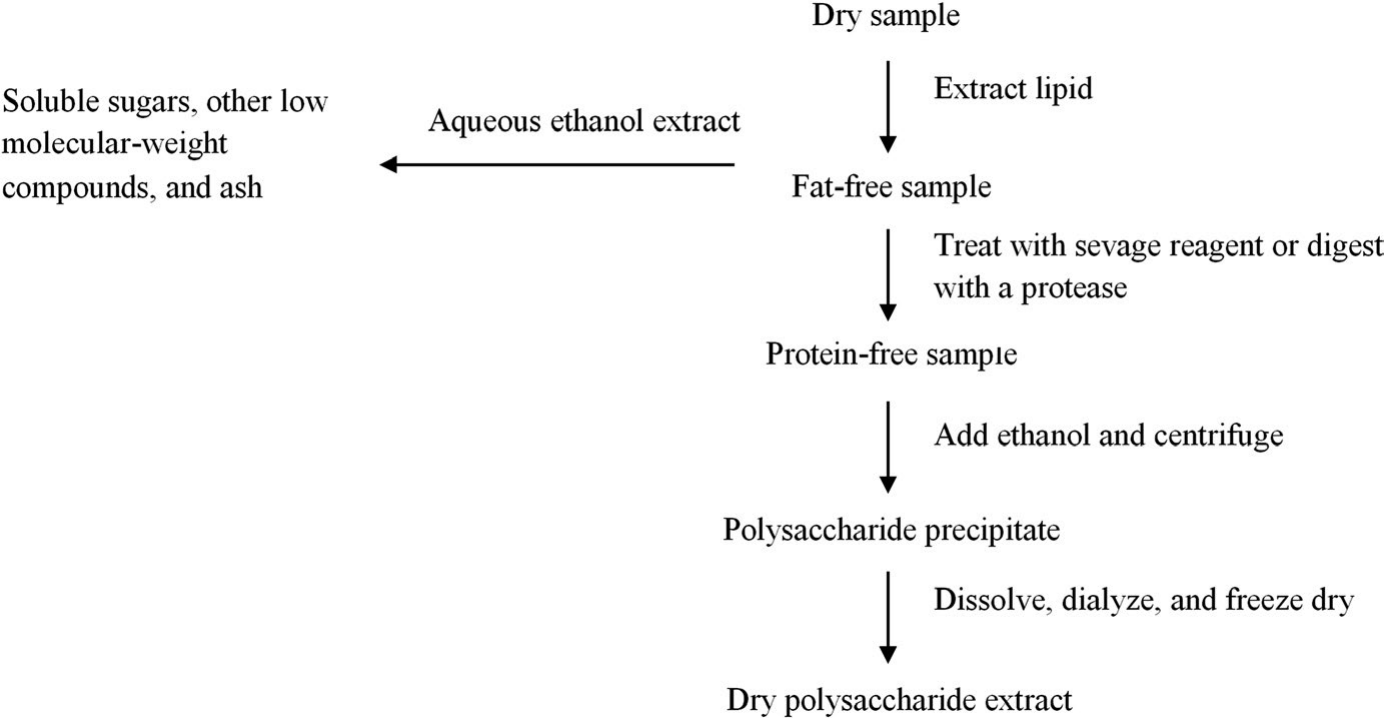
Flow diagram for purification of polysaccharides

Purification process is commonly performed as the primary step in the characterizing and exploring the potential application of polysaccharides in food and pharmaceutical systems (Cui, 2005). The elimination of proteins from the structure of gums is important since it leads to improving their thickening ability (Burkus & Temelli, 1998). Furthermore, it has been reported that the presence of protein in the polysaccharides structure can induce an inflammatory response in tissues that may limit the biological utilization of the polysaccharides (Tučková et al., 2002). Figure 1 presents the scheme for purification of water‐soluble polysaccharides.

It is often difficult to extract polysaccharides when oils, fats, and proteins are present. Therefore, lipid‐soluble substances should be removed first (Nielsen, 2010). Plant materials are usually defatted by dispersing in hot aqueous ethanol (80%–90% (v/v)) and washing the residue with absolute ethanol and acetone or ether (Andersson et al., 2006). Proteins can be removed using Sevag method (Staub, 1965), or by enzyme‐catalyzed hydrolysis. Any solubilized polysaccharides are precipitated by addition of four volumes of absolute ethanol (to give an ethanol concentration of 75%) to the cooled dispersion (Cui, 2005). The mixture is centrifuged, and the precipitate of water‐soluble polysaccharides is dialyzed and freeze‐dried (Stephen & Phillips, 2016).

The exudate gums are commonly obtained from the trunks and branches of trees in place of mechanical and microbial injuries (Phillips & Williams, 2000). The selection of efficient solvent to purify the exudate gums is different from species to species. In a recent study conducted by Fathi et al. (2016b), the effect of solvent type (acetone, methanol, and ethanol) and centrifugation process on yield, consistency coefficient (as a measure of total carbohydrate content), and lightness of PAGE were evaluated (Table 1). These authors reported that the precipitation of PAGE by acetone after centrifugation process led to producing the gums with maximum yield (51.3%), consistency coefficient (1.51 Pa s^n^) and lightness (74.35). However, the obtained yield of PAGE was less than that reported for *Prunus amygdalus* gum (58.2%), extracted using 4% H_2_O_2_ and 2 N NaOH (Bouaziz et al., 2015). The increase in lightness after the purification process is probably due to the elimination of coloring agents that were naturally present in the composition of crude hydrocolloids.

**TABLE 1:** The value of consistency index (Pa s^n^) obtained from power‐law model with concentration of 8% (w/w), purification yield, and lightness^†^

Solvent	Centrifuge (g)	Purification yield (%)	Consistency index (Pa s^n^)	Lightness
–	–	–	0.37 ± 0.03^e^	71.46 ± 0.50^d^
Methanol	–	26.1 ± 2.2^e^	0.39 ± 0.07^e^	71.68 ± 0.19^d^
Ethanol	–	18.0 ± 1.3^d^	0.47 ± 0.05^d^	71.88 ± 0.21^d^
Acetone	–	47.1 ± 2.2^a^	1.09 ± 0.11^b^	73.74 ± 0.07^b^
–	3,000	34.3 ± 2.8^b^	0.80 ± 0.04^c^	72.23 ± 0.12^c^
Methanol	3,000	25.6 ± 3.1^c^	0.78 ± 0.07^c^	72.27 ± 0.08^c^
Ethanol	3,000	25.5 ± 1.0^c^	1.12 ± 0.12^b^	73.97 ± 0.18^a^
Acetone	3,000	51.3 ± 3.1^a^	1.51 ± 0.08^a^	74.35 ± 0.56^a^

^†^ The same letter in each column is not significantly different at the 5% level.

## 3 CHEMICAL AND STRUCTURAL PROPERTIES

The chemical characterization, such as carbohydrate composition and content, is commonly used to evaluate the purity of gums (Chaplin & Kennedy, 1994; Cui, 2005). Furthermore, some chemical components have a considerable impact on functional properties of gums. The compositional properties of PAGE, compared to other *Prunus* species gums, are summarized in Table 2. PAGE is mainly composed of carbohydrate. Comparatively, the carbohydrate content obtained by Hamdani et al. (2017) was considerably greater than that reported by Fathi et al. (2016b). The method of gum purification has not been reported in Hamdani et al. (2017) research. The observed differences are most likely due to different environmental growing conditions, time of collection, source, age of tree, and contamination of exudate gums (Fathi et al., 2016a). An increased the carbohydrate content indicates a high level of purity. Therefore, it seems that PAGE purified by centrifugation process followed by acetone precipitation could not sufficiently improve the purity of this gum. However, further analyses such as gel permeation chromatography should be conducted to confirm this.

**TABLE 2:** Chemical properties of *Prunus armeniaca* gum, *Prunus cerasus* gum, *Prunus cerasoides* gum, *Prunus dulsic* gum, *and Prunus amygdalus* gum

Composition (%)		PAGE			*Prunus cerasus* gum	*Prunus cerasoides* gum	*Prunus dulsic* gum	*Prunus amygdalus* gum
		Fathi et al. (2016b)	Hamdani et al. (2017)	Jamila et al. (2020)	Fathi et al. (2016a)	Malsawmtluangi et al. (2014)	Mahfoudhi et al. (2012)	Bouaziz et al. (2015)
Total protein		2.91 ± 0.20	2.25 ± 0.03		2.40 ± 0.1	2.33 ± 1.25	2.45	–
Total carbohydrate		66.89 ± 0.02	83.08 ± 0.47		71.51 ± 0.02	73.72 ± 2.24	92.6	98.4
Uronic acid		10.47 ± 0.18	–		7.31 ± 0.54	11.05 ± 1.34	5.97	11
Ash		4.00 ± 0.05	3.67 ± 0.02		2.8 ± 0.01	2.55 ± 0.66	2.30	0.5
Moisture		6.9 ± 0.10	11.12 ± 0.01		6.20 ± 0.15	9.25 ± 0.54	2.04	1.1
Fat		1.59 ± 0.06	1.10 ± 0.20		1.48 ± 0.13		0.85	–
Crude fiber		–	1.88 ± 0.03		–		–	–
Monosaccharides	Arabinose	41.52 ± 0.67	–		31.31 ± 0.39	60.99 ± 0.34	46.82	26
	Galactose	23.72 ± 1.21	–		24.04 ± 0.89	5.44 ± 0.25	35.49	45
	Xylose	17.82 ± 0.19	–		21.31 ± 0.21	1.16 ± 0.11	10.90	7
	Mannose	14.40 ± 0.47	–		17.63 ± 0.96	–	0.75	10
	Rhamnose	2.54 ± 0.12	–		5.69 ± 0.69	1.37 ± 0.23	0.83	1
Elements (ppm)	Calcium (Ca)	11,043.41 ± 1.62		9,754.3 ± 78.1	5,200.2 ± 0.97	40.06 ± 2.11	142.50	–
	Iron (Fe)	963.00 ± 0.21		348.4 ± 15.9	552.3 ± 1.70	2.62 ± 0.04	10.75	–
	Sodium (Na)	112.39 ± 1.12		2,079.9 ± 52.8	264.3 ± 1.40	4.10 ± 0.29	12.50	–
	Phosphorus (P)	424.00 ± 1.32		223.2 ± 1.64	1,676.5 ± 1.32	–	–	–
	Selenium (Se)	<MDL^a^		0.007 ± 0.004	<MDL	<MDL	–	–
	Magnesium (Mg)	3,556.60 ± 1.97		2,506.7 ± 26.2	2,840.0 ± 4.00	–	300.00	–
	Manganese (Mn)	30.71 ± 0.00		ND^b^	19.2 ± 0.00	2.39 ± 0.019	–	–
	Potassium (K)	245.80 ± 0.26		7,305.5 ± 57.9	204.0 ± 1.00	9.80 ± 0.16	112.30	–
	Zinc (Zn)	198.92 ± 0.06		4.36 ± 0.900	106.8 ± 0.10	0.376 ± 0.03	1.50	–
	Cobalt (Co)	<MDL		0.151 ± 0.009	<MDL	–	–	–
	Chromium (Cr)	0.61 ± 0.00		0.908 ± 0.146	3.7 ± 0.00	–	–	–
	Copper (Cu)	0.25 ± 0.00		2.69 ± 0.070	0.5 ± 0.00	0.228 ± 0.01	3.31	–
	Tin (Sn)	<MDL		ND	<MDL	–	–	–
	Silver (Ag)	<MDL		ND	<MDL	–	–	–
	Aluminum (Al)	10.38 ± 0.02		300.4 ± 29.8	8.38 ± 0.50	–	–	–
	Nickel (Ni)	0.04 ± 0.00		3.38 ± 0.333	0.03 ± 0.00	–	–	–
	Lead (Pb)	<MDL		0.306 ± 0.030	<MDL	–	–	–
	Mercury (Hg)	<MDL		ND	<MDL	–	–	–
	Cadmium (Cd)	<MDL		0.003 ± 0.0005			–	–
	Arsenic (As)	<MDL		0.028 ± 0.0008				–

^a^ Method detection limit.

^b^ Not determined.

The monosaccharide composition of hydrocolloids is very important because it can affect their rheological and functional properties (Cui et al., 1996). The monosaccharide constituent of PAGE is summarized in Table 2. According to Zitko et al. (1965), PAGE is a polysaccharide containing xylose (Xyl), L‐arabinose (L‐Ara), and D‐galactose (D‐Gal) with a respective ratio of 1:8:8, with trace amounts of mannose (Man), D‐glucuronic (D‐GlcA), and 4‐*O*‐methyl‐D‐glucuronic acid (4‐*O*‐Me‐D‐GlcA). Chemical composition of PAGE exudates in response to infection of the tree or application of 2‐methyl‐4‐chlorophenoxyacetic acid was analyzed by Rosik et al. (1968). They indicated that the gums obtained after infection by *Monilia laxa*, *Monolia fructigena,* and chemical treatment were polysaccharides composed of D‐GlcA, 4‐*O*‐Me‐D‐GlcA, D‐Man, D‐Gal, D‐Xyl, and L‐Ara with ratios of 5:5:2:40:4:32, 5:5:3:35:4:25, and 5:5:2:26:5:17, respectively.

In another study, Fathi et al. (2016b) found that this gum is composed of Ara (41.52%), Gal (23.72%), Xyl (17.82%), Man (14.40%), and rhamnose (Rha; 2.54%). Since arabinose and galactose are the most abundant monosaccharides in the PAGE composition, it is suggested that this gum has an galactoarabinan‐like structure. In a recent study, Babken et al. (2018) used ^1^H and ^13^C NMR analyses to elucidate the structure of PAGE. They found that this gum is a complex polysaccharide composed of α and β‐L‐Ara*p*, α and β‐D‐Gal*p* and α and β‐D‐glucopyranoses (β‐D‐Glc*p*).

The comparison between the results of the monosaccharide composition analysis of the PAGE and those of reported by Simas et al. (2008) for *P*. *persica* gum revealed some discrepancies. These authors represented that *P. persica* gum was composed of Ara, Xyl, Man, Gal, and uronic acids in a molar ratio of 36:7:2:42:13. In addition, PAGE had different molar ratios of monosaccharide compared with those reported by Fathi et al. (2016a) for *Prunus cerasus* (5.5:4.2:3.8:3.1:1.0 for Ara, Gal, Xyl, Man, and Rha, respectively), by Malsawmtluangi et al. (2014) for *Prunus cerasoides* (52.3:4.7:1.0:1.2 for Ara, Gal, Xyl, and Rha, respectively), by Mahfoudhi et al. (2012) for *Prunus dulcis* (62.3:47.3:14.5:1.0:1.1 for Ara, Gal, Xyl, Man, and Rha, respectively) and by Bouaziz et al. (2015) for *P. amygdalus* (26.0:45.0:7.0:10.0:1.0 for Ara, Gal, Xyl, Man, and Rha, respectively).

**FIGURE 2:**
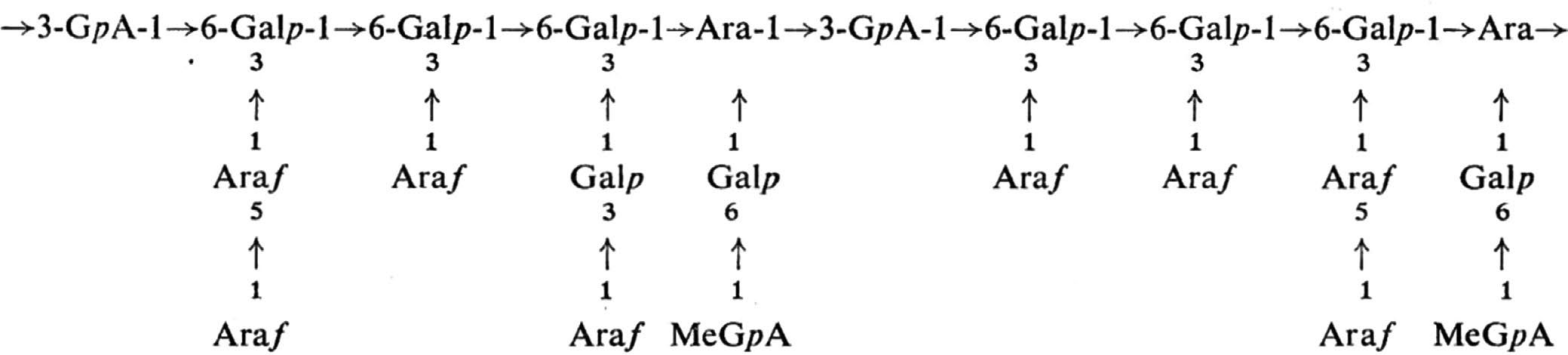
The proposed structure of *Prunus armeniaca* gum exudates

A research has provided more detailed structure information about PAGE was conducted for the first time by Zitko et al. (1965). These authors used chemical methods, such as partial acid hydrolysis and periodate oxidation, and proposed that PAGE has a backbone of β‐D‐Glc*p*A‐(1→6)‐β‐D‐Gal*p*‐(l→6)‐β‐D‐Gal*p*‐(l→6)‐D‐Gal and D‐Glc*p*A‐(l→2)‐Man‐(l→2)‐Man‐(l→Man units, which separated by L‐Ara*f* residues. The branches were mainly composed of 4‐*O*‐Me‐β‐Glc*p*A‐(l→6)‐D‐Gal units and L‐Ara*f* residues (Figure 2).

This structure was confirmed by Stephen and Shirley (1986) who used Smith degradation technique and methylation analysis to elucidate the PAGE structure. They showed that PAGE composed of glucuronic acid, mannose and galactose, and erythronic acid linked through glycolaldehyde.

According to Table 2, PAGE contains 6%–12% uronic acids. Uronic acids are anionic components that are both carbonyl and carboxylic acid groups (Linhardt et al., 1991). The presence of these components in polysaccharide composition is an indicator of their acidic nature (Ueno et al., 2019). Acidic polysaccharides tend to interact with oppositely charged macromolecules, and at pHs below their dissociation constant, the carboxyl groups will be dissociated, subsequently making it negatively charged (Sherahi et al., 2018). The negatively charged polysaccharides can be used as a carrier for encapsulation of ingredients (Gbassi & Vandamme, 2012; Liu et al., 2008; Solghi et al., 2020). For instance, in coacervation technique, the electrostatic interaction between negatively charged polymers, for example, PAGE and positively charged polymers/ions, leads to the formation of a coacervate that can entrap the ingredients. Moreover, it has been reported that the charged polymers have greater solubility than neutral ones (Hu & Goff, 2018), demonstrating that the solubility of PAGE is more than neutral polymers. However, further experiments should be carried out to confirm it.

Literature reviews (Table 2) show that PAGE has 1%–3% protein content (Table 2), evaluated by the Kjeldahl method. The protein molecular fraction of PAGE should be purified by Hydrophobic Interaction Chromatography as described previously by Renard et al. (2006) to show that this protein is part of the polysaccharide, as Arabic gum. The presence of protein in the PAGE composition could be also confirmed by FT‐IR analysis (Fathi et al., 2016b). The existence of proteins in the polysaccharide structure has determinant influence on their physical and functional properties (Choi et al., 2010; Lan et al., 2020). For instance, the gums containing proteins such as gum Arabic can be used as an emulsifier in food formulations (Salehi et al., 2019). Accordingly, PAGE can be employed as an emulsifier in food and pharmaceutical industries. The emulsifying capacity of PAGE will be discussed in the following sections.

As presented in Table 2, PAGE has 0.5%–4% ash. This value is in the range of ash content of hydrocolloids reported in the literature. According to the available literature, exudate gums have considerable amount of metal ions and neutralized cations (Jamila et al., 2020; Pachuau et al., 2012). These ions can change the physical and functional properties of the gums. For instance, gelling and viscosifying capacities of the gums depend on their mineral composition (Sherahi et al., 2017). The mineral constituents of PAGE were determined by some authors (Fathi et al., 2016b; Jamila et al., 2020). Table 2 summarizes these results. Fathi et al. (2016b) indicated that PAGE had high level of nutrients, especially calcium (11,043.41 ppm), magnesium (3,556.60 ppm), iron (963.00 ppm), phosphorus (424.00 ppm), and potassium (245.80 ppm). Jamila et al. (2020) reported that nutrient levels of PAGE were in the order of Ca>K>Mg>Na>Fe>P. By comparing these results with the results from Fathi et al. (2016b), it was found that there is inconsistency in the content (Table 2), which may be due to geographical variation. However, the nutrients values of PAGE were higher than most of commercial gums (Fadavi et al., 2014; Jahanbin et al., 2012; Mahfoudhi et al., 2012; Mohammad Amini & Razavi, 2012; Yebeyen et al., 2009), and thus, this gum can be added to food products to enrich their nutrient value.

The molecular weight of polymers is a key factor in predicting their functional properties (Phillips & Williams, 2000). For example, the gelation‐ and viscosity‐enhancing abilities of hydrocolloids mainly depend on their molecular weight. In general, following an increase in molecular weight of hydrocolloids, their viscosity increases (Nakayama, 1999; Liang et al., 2006). Furthermore, it has been reported that the polysaccharides with high molecular weight do not have tendency for absorption at the interface of water–air, and thus can be employed as stabilizer for protein foams (Martinez et al., 2005). Fathi et al. (2016b) reported that weight average molecular weight of PAGE was 5.69 × 10^5^ g/mol. This value of molecular weight was higher than those obtained previously by Zitko et al. (1965) (1.92 × 10^5^ g/mol), by Rosik et al. (1968) (0.92–1.37 × 10^5^ g/mol), and by Stephen and Shirley (1986) (1.50 × 10^5^ g/mol). This probably resulted from different determination methods. The molecular weight value of PAGE is close to *P. cerasoides* gum (5.50 × 10^5^ g/mol; Malsawmtluangi et al., 2014), and lower than the data reported for summer *Prunus avium* gum (2.99 × 10^6^ g/mol; Amarioarei et al., 2012), *P. cerasus* gum (1.12 × 10^7^ g/mol; Fathi et al., 2016a; Simas‐Tosin et al., 2009), and *Prunus persica* gum (5.61 × 10^6^ g/mol).

## 4 RHEOLOGICAL PROPERTIES

### 4.1 Dilute solution behavior

Evaluation of dilute solution behavior of the hydrocolloids provides data on their fundamental properties (Mays & Hadjichristidis, 1991). Various factors may affect the dilute solution behavior of biopolymers and consequently change their conformation and molecular properties (Rochefort & Middleman, 1987). Fathi et al. (2017) evaluated the effect of various ions, temperatures, and sugars on dilute solution properties of PAGE.

#### 4.1.1 Effect of temperature

The influence of temperature on dilute solution behavior of PAGE (at pH 7.0) was investigated by Fathi et al. (2017). They used five most common models including Kraemer, Huggins, Tanglertpaibul–Rao's, and Higiro 1 & 2 models to estimate the intrinsic viscosity of the gum.

According to Kraemer's empirical equation, intrinsic viscosity [*η*] can be computed from the extrapolation of ln *η*_rel_/*C* to infinite dilution (Kraemer, 1938):

(1) $$\frac{\ln\eta_{\text{rel}}}{C}=\left[\eta \right]+k_{k}{\left[\eta \right]}^{2}C$$

In Huggins equation (Huggins, 1942), *η* can be quantified from the extrapolation of *η*_sp_/*C* to zero concentration:

(2) $$\frac{\eta_{\text{sp}}}{C}=\left[\eta \right]+k_{H}{\left[\eta \right]}^{2}C$$

In the above equations, *k_k_*, *k_H_*, and *C* are Kraemer's constant, Huggins constant, and the concentration of gum, respectively.

In several researches, it has been demonstrated that the equations in which η is computed by determining the slopes of plots are more efficient for intrinsic viscosity determination than those obtained from intercepts of plots (Behrouzian et al., 2014; Hesarinejad et al., 2015; Mirabolhassani et al., 2016; Razavi et al., 2012; Yousefi et al., 2014). Accordingly, the values of relative viscosity or specific viscosity versus polymer concentration were plotted, and then, the slope of the plot was used to calculate *η*:

Tanglertpaibul–Rao's equation (Tanglertpaibul & Rao, 1987):

(3) $$\eta_{\text{rel}}=1+\left[\eta \right]C$$

Higiro's equations (Higiro et al., 2006):

(4) $$\eta_{\text{rel}}{\;\text{= e}}^{[\eta ]C}$$

(5) $$\eta_{\text{rel}}=\frac{1}{1‐[\eta ]C}$$

The results revealed that the values of determination coefficient (*R* ^2^) for all the models used were more than .89, exhibiting the appropriateness of these equations for determination of intrinsic viscosity at various temperatures. However, they reported that Tanglertpaibul–Rao's model had the highest *R* ^2^ and the lowest root mean square error (RMSE). Thus, this equation was introduced as the best model to describe the dilute solution properties of PAGE at tested temperatures (Fathi et al., 2016b, 2017).

The literature review showed that when the solution temperature increased, the values of intrinsic viscosity decreased. This trend is probably due to the reduction in the stability of hydrogen binding between PAGE macromolecules and the molecules of water, and also, on the reinforcement of the interaction stability between polymer chains (Razavi et al., 2012).

Fathi et al. (2017) used Arrhenius’ equation to evaluate the temperature dependency of PAGE solution in dilute regime (Draget, 2006):

(6) $$\eta =A\cdot e^{\frac{E_{a}}{R\cdot T}}$$

where *A*, *η*, *R*, *E_a_*, and *T* are constant numbers, the dynamic viscosity, the universal gas constant (8.314 kJ/kg mol K), the activation energy (kJ/kg mol), and the absolute temperature (K), respectively.

When the intrinsic viscosity is used instead of dynamic viscosity, the calculated slope of the resulting plot can be used to determine the chain flexibility of biopolymer macromolecules in the solution (Fathi et al., 2017). The values of *E_a_*/*R*, known as chain flexibility factor and activation energy for PAGE, were 997.3 (1/K) and 0.83 × 10^7^ (J/kg mol), respectively. The chain flexibility factor of PAGE is close to that obtained for Balangu seed gum (1,156.53 1/K; Amini & Razavi, 2012), exhibiting a similar chain flexibility for their macromolecules. Furthermore, due to low value of activation energy obtained for PAGE than most of hydrocolloids such as Balangu seed gum (1 × 10^7^ J/kg mol; Amini & Razavi, 2012), chitosan (1.5 × 10^7^ J/kg mol; Wang & Xu, 1994), and sage seed gum (2.53 × 10^7^ J/kg mol; Yousefi et al., 2014), it can be concluded that PAGE has low‐temperature dependency. Overall PAGE can be used as a food additive for application in food processing that require high‐temperature stability.

#### 4.1.2 Effect of ion type and ion concentration

As mentioned above, PAGE has a polyelectrolyte nature, and thus, it is expected that its macromolecular conformation changes in the presence of cations. Fathi et al. (2017) evaluated the impact of ion type (NaCl and CaCl_2_) and ionic strength on the dilute solution properties of PAGE. Higiro 1 model was the best for describing the rheological behavior of PAGE in dilute regime and showed maximum and minimum *R* ^2^ and RMSE, respectively. The authors observed that following an increase in ionic strength, the intrinsic viscosity of PAGE decreases. This decreasing trend has been attributed to the increase in shielding charges at higher ion concentration, which results in the contraction of the molecules, and decrease in intrinsic viscosity (Amini & Razavi, 2012). The impact of CaCl_2_ addition on reduction in intrinsic viscosity was more pronounced than NaCl. Due to the presence of uronic acids in PAGE composition, CaCl_2_ ions can tend to take part in the formation of coacervate and thus result in more extensive molecular contraction (Kök et al., 2009).

#### 4.1.3 Effect of sugar type and sugar concentration

Dilute solution properties of gums such as viscosity also depend on sugar concentration (Cui & Wang, 2005). Fathi et al. (2017) evaluated the effect of sugar type (lactose and sucrose) and sugar concentration on dilute solution properties of PAGE and found that the best model for determination of intrinsic viscosity at all sugar types and sugar concentrations was Higiro 2. When the sugar concentration increased, the value of intrinsic viscosity showed a decreasing trend and this effect was more pronounced for lactose than sucrose. This decreasing effect has been associated with the increase in competition between PAGE and sugar for interaction with water with elevation of sugar concentration which leading to decrease in availability of water molecules for interaction with the biopolymer (Durchschlag, 1989).

### 4.2 Steady‐state properties

Steady shear measurements are broadly carried out to evaluate the potential application of gums as thickener and stabilizer (Williams & Phillips, 2004). Various factors such as gum concentration, solution temperature, ion and sugar addition, irradiation, and ultrasonic treatment can affect the viscosifying ability of hydrocolloids (Marcotte et al., 2001; Marcotte et al., 2001; Zendeboodi et al., 2019).

#### 4.2.1 Effect of gum concentration

The influence of gum concentration on steady shear behavior of PAGE was evaluated by Fathi et al. (2016b). They recorded the values of shear stress against shear rate in the range of 14–300 s^−1^. The tests were carried out using a rotational viscometer equipped with a heating circulator. They used C25 spindle for the rheological evaluation.

The steady shear evaluation at low shear rate provides useful information on the consistency of products in the mouth (Morris, 1990). On the other hand, the data obtained from steady shear measurements at high shear rate are useful for predicting the behavior of the gums in operations such as pumping fluids (Anvari et al., 2016). Since the steady shear behavior of PAGE was only investigated in high shear rate, the resulting data can be used for predicting the behavior of the gum in food processing operations. Additionally, the authors only used power‐law equation to describe the steady shear behavior of PAGE. Further rheological studies can be conducted on the evaluation of steady shear behavior of PAGE with other models such as Casson, Herschel Barkly, and Sisko as well as investigation of time‐dependent behavior of this gum.

Moreover, Fathi et al. (2016b) observed that the values of flow behavior indices in all the tested solutions were below one, revealing shear thinning behavior of PAGE solutions. This behavior has arisen from its polymeric structure and high molecular weight. When gum concentration increased, the value of flow behavior index decreased, demonstrating more pseudoplasticity at higher gum concentration. With increase in gum concentration, the values of consistency coefficient increased showing that the thickening ability of the gum improved. This effect is due to the increase in solid content at higher gum concentration that increases molecular entanglements and as a result improves the thickening ability of the gum (Maskan & Göǧüş, 2000).

#### 4.2.2 Effect of solution temperature

Due to the extensive range of temperatures encountered over food operations, the temperature sensitivity of gums should be evaluated (Wu et al., 2015). As reported by Fathi et al. (2016b), the gum solutions at various temperatures (10, 20, 30, and 40°C) had a non‐Newtonian shear thinning behavior. As expected, the authors reported that when the solution temperature increased, the flow behavior index increased, indicating a tendency to Newtonian behavior at high temperatures. However, an increase in solution temperature resulted in a decrease in consistency coefficient. This decreasing effect can be considered as an advantage when the gum is used in high shear processing operations such as pumping (Fathi et al., 2016b). The decrease in consistency coefficient (as an indication of viscosity) is related to the increase in molecular mobility, and in turn decrease in flow resistance (Karazhiyan et al., 2009). With increase in the concentration of PAGE solution, the values of *E_a_* declined, revealing lower temperature sensitivity at higher solution concentration.

### 4.3 Dynamic rheological properties

Dynamic rheometry measurement has been employed by many scientists to obtain valuable data on the viscoelastic properties of biopolymers without cleaving their structural elements (Gunasekaran & Ak, 2000). This experiment permits researchers to relate dynamic rheological parameters to the molecular structure properties of the gum solution systems (Choi et al., 2006).

#### 4.3.1 Strain and frequency sweep tests

Hamdani et al. (2017) investigated viscoelastic properties of PAGE dispersions (1% w/v). The sample preparation was carried out at 90°C. At high temperatures, polysaccharides may be degraded, and hence, it would have been better if this test was carried out at lower temperatures.

Before carrying out a frequency sweep experiment, a strain/stress sweep test must first be done to determine the critical strain. Critical strain is defined as the maximum deformation that a system can withstand without structural collapse. Hamdani et al. (2017) demonstrated that the linear viscoelastic range of the sample was 1 Pa, and hence, frequency sweep test was conducted at this region. It was found that in the range of evaluated frequency, the value of loss modulus (*G*″) was always superior to the storage modulus (*G*′), showing viscose nature of this gum.

#### 4.3.2 Effect of gamma irradiation

Effect of various doses of gamma irradiation on viscoelastic properties of PAGE solution was evaluated by Hamdani et al. (2017). They reported that when the irradiation dose increase from 0 to 2.5 kGy, no change was observed in the viscosity of the sample. But, when the irradiation dose reaches to 5 kGy, a profound decrease in the viscosity of PAGE solution was observed. This decreasing trend has been attributed to the reduction of the area swept by molecules at higher irradiation dose which results in the decrease in viscosity.

## 5 PHYSICAL PROPERTIES

### 5.1 Flow properties

Flow properties such as bulk and tapped densities and repose angle are determinant factors for application of powders in food and pharmaceutical systems (Malsawmtluangi et al., 2014). Hamdani et al. (2017) reported that the values of tapped and bulk densities of PAGE were 0.66 and 0.82 (g/ml), respectively. The values of tapped density of PAGE were slightly greater than the data reported for other Rosaceae family gum exudates like *Prunus dulsis* (0.517 g/ml; Farooq et al., 2014). Moreover, the bulk and tapped density values of PAGE increased upon exposure to gamma irradiation (Hamdani et al., 2017).

Hausner ratio and Carr's index are two parameters that have been broadly employed to estimate the flow behavior of powders (Emery et al., 2009). The Hausner ratio is a measure of interparticle friction, whereas Carr's index is a measure of the potential powder arch or bridge strength and stability (Kumar et al., 2001). It has been reported that for powders with poor flow property, the value of Hausner ratio is >1.25; however, powders with good flowability have a Hausner ratio value lower than 1.25 (Wells, 1988). Hamdani et al. (2017) demonstrated that PAGE had Hausner ratio of 0.80, exhibiting good flowability for this exudate gum. This value is lower than those reported for other exudate gums like *P. cerasoides* (1.33; Malsawmtluangi et al., 2014) and *P. dulcis* (1.93; Farooq et al., 2014). Accordingly, a better flowability is highly expected for PAGE than these gums.

Carr's index value can be used to categorize the flowability of powders: 5–15 (excellent), 15–16 (good), 18–21 (fair), and 23–28 (poor flow properties; Carr, 1965). According to the literature described by Hamdani et al. (2017), the value of this parameter for PAGE powder was 19.38%, denoting that PAGE had fair flow characteristics. These authors also indicated that when PAGE was treated with gamma irradiation, Carr's index value decreased by 12.74%. It is evident that treated samples showed excellent flow properties.

It should be emphasized that the flowability of powders is a complex phenomenon and several factors such as powder properties and physicochemical characteristics of the system may affect the properties of the powder flow system (Kim et al., 2005). Some of these factors are summarized in Table 3. Particle size is one of the most important factors affecting powder flowability, and increasing this factor improves the flowability of powders. Solid particle flow is a complex interaction among particle size, shape, and density.

**TABLE 3:** Some of parameters affecting the flowability of powders

Particle properties	Intrinsic factors	External factors
Chemical composition	Temperature	Feeding rate
Density	Air relative humidity	Vibration
Size	Compaction level	Hopper dimension
Shape	Coating, Agglomeration	Discharge aid
Roughness	Segregation	
Friction	Anticaking agents	
Particle compressibility (Hardness, elasticity, ductility)		
Conductivity, capacitance, propensity to electrostatic charge)		

## 6 FUNCTIONAL PROPERTIES

### 6.1 Emulsion capacity and stability

Most of hydrocolloids are used to control the emulsion shelf life (Dickinson, 2009). Generally, to test the efficiency of gums as emulsifier, the gum concentration required to obtain an emulsifying system with the lowest droplet size is measured (Dickinson, 1988). Based on physical law, contact angle between 0° and 45° means a hydrophilic environment, which increases dispersive phase sedimentation (Chen, 1988; Staicopolus, 1962). Chichoyan (2015) specified the contact angle of PAGE water solutions to elucidate the concentration needed for the gum can act as stabilizer. The contact angles of the gum solution with concentration of 5%–15% were <45°. But, for the gum with higher concentration (20%), the contact angle value exceeded 45°, indicating 20% of PAGE concentration is the starting minimal concentration for colloidal system stabilization.

#### 6.1.1 Effect of gamma irradiation

The influence of gamma irradiation on emulsion capacity of PAGE was investigated by Hamdani et al. (2017). Emulsion capacity of PAGE was 24.33% which increased up to 24.75% when the gamma irradiation was increased to 5 kGy. The suitable stabilizing ability of polysaccharides is commonly associated with their high molecular weight and gelation capacity (Liu et al., 2019). Similarly, a slight increase in emulsion stability was also observed with increase in gamma irradiation. The improved emulsion stability and capacity in the exposure of gamma irradiation can be related to the cleavage of glycoside interaction of polysaccharides that increases the exposure of both hydrophilic and hydrophobic groups.

#### 6.1.2 Effect of polymer ratio

The influence of PAGE/apple pectin complex as emulsifier on nano‐emulsion stability was evaluated by Shamsara et al. (2017). They determined creaming index, droplet size, and zeta potential and found that when the complex formed by PAGE/apple pectin in the ratio of 21.4:1 was used as emulsifier, the obtained values for maximum creaming index, zeta potential, and droplet size ere most stable. Their results revealed that PAGE/pectin with the ratio of 21.4 (W/W) showed the best creaming stability (CI ~ 84%) after 10 days.

#### 6.1.3 Effect of pH change

The impact of pH (2, 3, 4, 5, 6, and 7) on the stability of PAGE–lactoglobulin two‐layer nano‐emulsions was investigated by Shamsara et al. (2015). The lowest and highest diameter of particle size was observed at pH of 4 and 7, respectively. The surface charge of particles is a key factor that determines the emulsion stability. The authors reported that at pH 4, PAGE, and lactoglobulin had oppositely charge; thus, this pH was introduced as optimum pH to achieve an emulsion with the best stability. The influence of pH changes on the emulsifying ability of PAGE/apple pectin complex was also tested by Shamsara et al. (2017). The authors indicated that when the pH was increased from 2 to 7, the droplet size of prepared emulsion significantly increased from 763 to 836 nm. Furthermore, it was also reported that following an increase in pH emulsion range 2–7, the zeta potential of the fabricated emulation decreased from −17 to −21 mV. This effect has been attributed to the presence of arabinogalactan proteins (AP) in PAGE structure which was confirmed by FT‐IR and compositional analyses described above. At pH below the isoelectric point of AP, like pH 2, some parts of AG and the whole of pectin have positive and negative charge, respectively, and the electrostatic interaction between them leads to improvement of the emulsion.

#### 6.1.4 Effect of ultrasonic treatment

The impacts of sonication time (0, 5, 10, 15, and 20 min) and amplitude (25%, 50%, 75%, and 100%) on the emulsifying ability of PAGE/apple pectin complex were analyzed by Shamsara et al. (2017). They found that when the long ultrasonic time with high voltage was employed, an emulsion with the smallest droplet size was obtained. Comparatively, the droplet size of ultrasonic‐treated emulsion was significantly smaller than control. Additionally, the emulsion stability of the treated emulsion over 10 days storage was more than the control. These authors also demonstrated that there was no profound difference between control sample and those treated with various ultrasound amplitudes. Overall, they reported that the fabricated emulsion treated with 10 min of ultrasonic treatment at an amplitude of 25% yielded optimum results in terms of droplet size, zeta potential, and creaming index.

#### 6.1.5 Effect of thermal treatment

The effect of temperature on creaming index, particle size, and zeta potential of prepared emulsion by PAGE/apple pectin complex was investigated by Shamsara et al. (2017). For this purpose, the prepared emulsions were incubated at 25, 37, 50, 60, and 80°C and their results showed that there is an increase in the droplet size of incubated emulsions with increase in temperature. This phenomenon is associated with the increase in kinetic mobility of polysaccharides at higher temperature which resulted in the formation of bigger droplets (Shamsara et al., 2017). Furthermore, the authors observed that with an increase in temperature, the negative charge decreases. On the other hand, the creaming index of the emulsion system increased as the temperature increased up to 60°C. However, with further increase in temperature, the creaming index decreased.

### 6.2 Antioxidant capacity

Various techniques are employed to determine antioxidant capacity of gums, and among them, DPPH scavenging assay is commonly used for this purpose. EC_50_, the concentration required for 50% inhibition, was used to estimate the antiradical activity of PAGE by Ergin et al. (2018). A smaller value of this parameter shows a more antiradical activity. The authors compared the values of EC_50_ to three exudate gums (PAGE, cherry gum, and gum Arabic). It was found that the EC_50_ values for the tested gums were as follows: PAGE˃cherry gum˃gum Arabic. Therefore, it can be concluded that the antiradical activity of PAGE is less than cherry gum and gum Arabic. In a different study, Jamila et al. (2020) reported that the scavenging activity of PAGE against DPPH radical was more than that of other gums such as *Salmalia malabarica*, *Acacia Arabica*, *Acacia modesta*, *Prunus persica,* and *Prunus domestica* gums. These authors demonstrated that the EC_50_ value of PAGE was 133.2 mg/ml. Polysaccharide interacts with free radicals like DPPH to produce products with high stability (Salehi et al., 2019). Due to the ability of phenolic compounds to donate hydrogen and stable radical intermediates, these compounds can inhibit oxidation of food products especially that of oil and fatty acids (Cuvelier et al., 1992; Maillard et al., 1996). Based on previous studies, there is a positive correlation between the amount of phenolic compounds and antioxidant activity of vegetables and fruits (Jayaprakasha et al., 2008; Kornsteiner et al., 2006; Li et al., 2009; Martínez et al., 2012). Total phenol content (TPC) of PAGE, cherry gum, and gum Arabic were as follows: PAGE ˂ cherry gum ˂ gum Arabic. The positive relation of phenolic compounds and antiradical activity also was confirmed in this study. Accordingly, it is clear that phenolic compound present in PAGE is strongly involved in antioxidant activity as determined by DPPH assay. Babken et al. (2018) determined the phenolic profile of PAGE using GC‐MS analysis. It was found that PAGE had, on average, 7.58% catechols, 4.27% hydroquinones, and 5.69% pyrogallol.

Hamdani et al. (2018) compared the antioxidant activity of PAGE to acacia and karaya gums. These authors used two solvents (ethanol and methanol) to compare the extractability of antioxidants between the solvents used. Their results showed that the total phenolic content (TPC) of both ethanolic and methanolic extracts of PAGE was higher than those of acacia and karaya gums. Moreover, TPC of methanolic extract of PAGE was higher than that of ethanolic extract, which is related to the variable solubility of phenolic compounds in tested solvents.

## 7 FOOD AND PHARMACEUTICAL APPLICATIONS

### 7.1 Food applications

The effect of edible coating based on PAGE containing *Satureja intermedia* extract with various concentrations (1%, 2%, and 3%) on antifungal and antioxidant properties of fresh wild almond (*Amygdalus scoparia*) kernels was investigated by Hashemi and Raeisi (2018). They observed that coating of the samples with the apricot gum containing the extract had considerable antifungal effect against *Aspergillus flavus*, *Alternaria alternate*, *Fusarium oxysporum,* and *Penicillium citrinum*. This effect was reinforced when the extract concentration increased. Furthermore, with an increase in extract concentration, the anti‐DPPH radical activity of the coating increased. Over the storage period (60 days), the coated samples had lower fungal contamination as compared to the control. Antioxidative analyses demonstrated that the amounts of oxidative compounds and fatty acid profile variation of the coated samples were lower than the control. The authors reported that the samples coated with 3% of the extract exhibited maximum antiradical and antifungal activities. In conclusion, it can be suggested that the developed coating can be utilized to extend the shelf life of fresh and dried fruit.

In another study conducted by Ergin et al. (2018), an edible film was developed to coat strawberry and loquat fruits. Thermal analysis demonstrated that the developed films were heat tolerant up to 400°C. The morphological characteristics of the films, analyzed by *SEM*, showed that the surface of the films was homogeneous with regular structure. The shelf life of the coated/uncoated fruits stored in refrigerator was compared in terms of shelf life, sensory, and microbiologic characteristics. The authors stated that the coating process led to extended shelf life of the fruits. From a microbiological point of view, the total bacteria, yeast–mold, and coliform bacteria of the control samples were higher than coated ones after the storage period. Furthermore, the coated samples had better organoleptic properties than the controls. Overall, Ergin et al. (2018) concluded that cherry gum and PAGE are suitable to coat fruits.

### 7.2 Pharmaceutical applications

Plant‐based gums have very wide applications in pharmaceutical science (Seyedabadi et al., 2020). They are mostly used as suspending, stabilizing, emulsifying, thickening, and binding agents and also as matrices for sustained release of drugs (Efentakis & Koutlis, 2001). Gums are interesting polymers because they often show unique biological and physicochemical activities at costs lower than synthetic polymers (Prajapati et al., 2013). Azam Khan et al. (2012) investigated the potential application of PAGE and *P. domestica* gum for their sustained release ability compared with hydroxypropyl methyl cellulose (HPMC). They showed that when PAGE and *P. domestica* gum were used in combination ratio of 1:1, release efficiency was improved and at optimum formulation, release profile was comparable to standard marketed formulation. These authors suggested that PAGE can be used as matrix former in tablet formulation. The synergistic binding potential of PAGE and *P. domestica* gum in tablet formulations was also investigated by Rahim et al. (2015) and Rahim et al. (2018). They reported that the gums used had better binding capacity for preparation of uncoated tablet dosage form than PVP K30. In a similar research, the gum binding features comparing gum Arabic and polyvinylpyrrolidone were studied (Şensoy et al., 2006). Results illustrated that PAGE is a promising pharmaceutical binder in tablet formulations.

Additionally, in a patent described by Rhodes (1989), it has been found that the release rate of ingredient in water‐soluble matrix may be decreased by changing the arrangement of the ingredients.

### 7.3 Application in corrosion inhibition

There are some researches that studied natural inhibitor substances as corrosion inhibitors for iron and steel materials in acidic media (Abdallah, 2004; El‐Etre, 2003; El‐Etre & Abdallah, 2000; Oguzie, 2005). Alwaan and Mahdi (2016) evaluated the effect of PAGE concentration and different temperatures (17–40°C) on the ability of this gum for corrosion inhibition of mild steel. It was found that with addition of PAGE to the acid solution (1 M HCl), the weight loss of the mild steel decreased and due to the presence of carbonyl and hydroxyl groups in PAGE composition, PAGE can adhere on the iron metal. At low temperature (17°C), applying PAGE had no effect on the weight loss of the steel. This effect may be due to low solubility of the polymer at low temperature.

### 7.4 Acting as organic additive in tissue culture media

In order to grow tissue, tissue culture media needs a supply of polysaccharides (Kozai, 1991). Commonly, some sugars such as sucrose, glucose, and sorbitol were added as carbon sources. To date, various studies have been carried out to evaluate the effect of adding new carbon sources on in vitro callus growth. In a recent study, Khorsha et al. (2016) investigated the usefulness of PAGE as an organic additive in the growth of carrot, stevia, and grapevine. With the incorporation of PAGE in the media, the fresh weight and volume increased and the pigmentation improved. Furthermore, with addition of PAGE, the shoot multiplication and rooting parameters in stevia and grapevine improved. Considering the mentioned positive effects, the application of PAGE in commercial tissue culture protocol is recommended.

### 7.5 Application to fabricate nanoparticles with biological activity

Synthesis of nanoparticles using natural products, such as gums, resins, and medicinal plants, is a succeeding area of research, and in recent years, there has been an upsurge in interest in the use of diverse natural products for the synthesis of metallic nanoparticles (Kumar & Yadav, 2009). In a study conducted by Islam et al. (2019), PAGE was used to synthetize gold and silver nanoparticles (Au‐ and Ag‐NPs) with diverse biological activity and high thermal stability. The biosynthesized Au and Ag nanoparticles had diameter range of 10–40 and 5–30 nm, respectively. The nanostructure of the fabricated particles was mostly spherical but a small number of anisotropic nanostructures such as nanotriangles. Disk diffusion test demonstrated that the developed nanoparticles had moderate antibacterial activity against *Staphylococcus aureus*, *Escherichia coli*, and *Pseudomonas aeruginosa*. It can be seen that the nanoparticles had a greater antimicrobial activity against *S. aureus*, as a gram‐positive bacteria than *E*. *coli* and *P. aeruginosa*, as gram‐negative strains. According to the literature, gram‐negative bacteria have a thin peptidoglycan layer, an outer and inner membrane that protects bacteria against antimicrobial agents (Ahmad et al., 2020; May & Silhavy, 2017; Narita, 2011; Tortora et al., 2004).

## 8 SUMMARY AND FUTURE TREND

In the present review article, the chemical composition, structure, rheological, and functional properties and potential application of PAGE as an attractive source of polysaccharide were reviewed. PAGE is a heterogeneous polysaccharide with an arabinogalactan structure. Due to its specific structure, it can be introduced as a suitable candidate for fiber formation using electrospinning technique. A future study is needed to evaluate the potential application of PAGE to the formation of nanofibers. PAGE has a polyelectrolyte nature, and thus can be used to encapsulate phytochemicals using coacervation method. To date, several studies have focused on the potential capacity of PAGE in food, pharmaceutical, and other industries; however, further studies should be carried out to explore other applications of this gum.

## Data Availability

Research data are not shared.
